# Remote Clinical Assessments and Management During COVID-19: Views of the Patients and Clinicians About the Future Preferences

**DOI:** 10.1192/bjo.2022.382

**Published:** 2022-06-20

**Authors:** Nilamadhab Kar, Lai-Ting Cheung, Stephen Jiwanmall

**Affiliations:** Black Country Healthcare NHS Foundation Trust, Wolverhampton, United Kingdom

## Abstract

**Aims:**

During the COVID-19 pandemic most clinical services changed to remote consultation and management to minimise virus transmission by direct contact. As the social distancing and restrictions have eased with greater control of the pandemic, the nature of consultations is going to change. At this juncture we intended to understand the perception and satisfaction of patients and clinicians on remote consultations and management during COVID-19 and to determine their preference about clinical engagement in the future.

**Methods:**

This was a trust-wide anonymous survey conducted through surveymonkey. It involved both patients and mental healthcare staff (MHS) and explored about the quality and satisfaction in remote consultations, option to patients, and use of remote consultations in future. Clinicians were sent the online link to complete, with a reminder two weeks later. The patients were explained during their appointments about the survey, those who agreed to participate and gave informed consent, their responses to the questions were recorded.

**Results:**

The sample consisted of 78 patients and 107 MHS representing adult, old age, children and adolescent and intellectual disability subspecialties. Most (92.4%) participants had participated in remote consultations and understood the reason behind it. Around a third (32.7%) of MHS and 46.2% of patients felt strongly satisfied in remote consultations, and together with satisfaction these were 56.1% v 71.8% respectively (p < 0.05). The quality of the remote consultations were considered somewhat (11.2% v 23.1%) or a lot better (8.4% v 15.4%) by MHS and patients respectively (p < 0.05). Majority (82.7%) felt that an option should be given to patients for the type of consultation, face to face or remote. After the pandemic, the preference for psychiatric consultations were primarily face to face (30.3%), primarily remote (8.6%) and a mixture of the two (61.1%); there were no difference between patients and MHS. However while 71.4% doctors, 70.8% other clinicians (occupational therapists and psychologists) and 75.0% of clinical managers opted for mixture of face to face and remote, 26.9% of nurses opted for that. Background subspecialty, age group, ethnicity, experience of remote consultation with GP or hospital doctors, attendance or admission to general or psychiatric hospitals during pandemic, disabilities, or having COVID-19 did not influence the suggestion for the future consultation type.

**Conclusion:**

Following the pandemic, both clinicians and patients express a preference for a mixture of face to face and remote consultations; and an option regarding that should be given to the patients.

